# Tracking between cardiovascular-related measures at 4 and 8 years of age in the INMA-Asturias cohort

**DOI:** 10.1007/s00431-023-05051-8

**Published:** 2023-06-20

**Authors:** Rocío Fernández-Iglesias, Pablo Martinez-Camblor, Ana Fernández-Somoano, Cristina Rodríguez-Dehli, Rafael Venta-Obaya, Margaret R. Karagas, Adonina Tardón, Isolina Riaño-Galán

**Affiliations:** 1 Spanish Consortium for Research On Epidemiology and Public Health (CIBERESP), Monforte de Lemos Avenue, 3-5, 28029 Madrid, Spain; 2 Unit of Molecular Cancer Epidemiology, University Institute of Oncology of the Principality of Asturias (IUOPA), Department of Medicine, University of Oviedo, Julian Clavería Street S/N, 33006 Oviedo, Asturias Spain; 3 Instituto de Investigación Sanitaria del Principado de Asturias (ISPA), Roma Avenue S/N, 33001 Oviedo, Asturias Spain; 4 Biomedical Data Science Department, Geisel School of Medicine at Dartmouth, Lebanon, NH USA; 5 Faculty of Health Sciences, Universidad Autonoma de Chile, 7500912 Providencia, Chile; 6 Servicio de Pediatría, Hospital San Agustín, Heros Street, 4, 33410 Avilés, Asturias Spain; 7 Servicio de Bioquímica, Hospital San Agustín, Heros Street, 4, 33410 Avilés, Asturias Spain; 8 Departamento de Bioquímica y Biología Molecular, University of Oviedo, Fernando Bongera Street, S/N, 33006 Oviedo, Asturias Spain; 9 Department of Epidemiology, Geisel School of Medicine at Dartmouth, Lebanon, NH USA; 10 Endocrinología Pediátrica, Servicio de Pediatría, HUCA, Roma Avenue S/N, 33001 Oviedo, Asturias Spain

**Keywords:** Cardiovascular risk, Childhood, Dyslipidemia, Hyperglycemia, Hypertension, Obesity, Quantile regression, Tracking

## Abstract

Identifying cardiovascular-related measures that track from early childhood into later ages may help inform early prevention targets for cardiovascular disease. In this study, the tracking of triglycerides (TG), high-density cholesterol (HDL-c), atherogenic coefficient (AC), waist circumference to height ratio (WC/Height), mean arterial pressure (MAP), and homeostatic model assessment of insulin resistance (HOMA-IR) was examined in the INMA-Asturias cohort between 4 and 8 years of age. The analysis was conducted in 307 children who participated in the INMA-Asturias cohort (Spain) at 4 and at 8 years of age. Quantile regression models were used to evaluate tracking between measures at both ages, with each measure at 8 years as the dependent variable and the rank transformation of the same measure at 4 years as the independent variable. We found a positive association between HDL-c rank at 4 years and higher quantiles of the HDL-c distribution at 8 years, with an increase of 2.93 mg/dL (95% CI: 1.98, 3.87) per decile in the 0.9 quantile. A positive association was also found for WC/Height, with an increase of 0.008 (95% CI: 0.004, 0.012) per decile in the 0.9 quantile. We observed that tracking for AC increased in the higher quantiles of the distribution at 8 years, with an increase of 0.11 (95% CI: 0.09, 0.14) in the 0.6 quantile compared to an effect of 0.15 (95% CI: 0.09, 0.21) in the 0.9 quantile.

*Conclusions*: Adult markers of dyslipidemia and central obesity tracked between ages 4 and 8 years. For AC, tracking increased in the higher quantiles of the distribution.

**Table:** 

**What is Known:** • *Atherosclerosis begins in early life, so preventive efforts that start in childhood may delay progression to clinical disease. Determine what cardiovascular risk factors track into time since childhood bring the opportunity to identified those subjects at risk for later cardiovascular disease.* • *The study of risk factors in health populations and, particularly in children, copes with not clear and/or controversial thresholds definition. This makes it challenging to study tracking in pediatric ages.*	
**What is New:** • *Quantile regression is a useful tool for assessing the tracking of risk factors for which there are no clinically meaningful thresholds. The increasing trend observed in the tracking of dyslipidemia suggests the possible difficulty that children with abnormal values at 4 years of age might have in normalizing them in future years.* • *The findings of this article may help to determine which cardiovascular-related measures could be screened and followed-up in children.*

**Supplementary Information:**

The online version contains supplementary material available at 10.1007/s00431-023-05051-8.

## Background

Abnormal values of cardiovascular-related measures are frequently detected in adulthood [1] but may also be present in childhood [2]. This does not increase the risk of cardiovascular diseases (CVDs) in childhood itself; children rarely experience cardiovascular diseases and these occurrences are mainly caused by congenital heart problems or genetic syndromes [3]. Atherosclerosis, one of the main CVD triggers [4] in adults, is an accumulative process that can begin in childhood and youth [5–7]. Therefore, researchers have been trying to answer whether those subjects exposed to specific metabolic alterations in childhood will have higher risk of developing CVDs — or early CVDs — in adulthood [8].

The study of the association between underlying cardiovascular disease indicators in childhood and CVDs in adulthood has been challenging due to the difficulty of following a young sample the time required to observe in this population CVD events. Several studies have shown evidence that CVDs are associated with childhood metabolic alterations [9]. For instance, a study of 38,589 participants aged 3 to 19 years from the USA, Finland, and Australia found an association between body mass index (BMI), systolic blood pressure, triglycerides, and cholesterol with cardiovascular events in midlife [10], and strongest associations with these factors in aggregate. These findings can be explained by a risk accumulation model, in which risk factors present at each life stage further increase risk in adulthood; a risk chain model, in which risk in childhood is mediated by risk in adulthood; or a sensitivity period model, in which exposure at a particular time in life course confers more risk compared with other stages [9, 11]. Under either scenario, identifying metabolic alterations that are more likely to track from childhood into future years will help inform targets of early prevention.

One of the main difficulties in tracking metabolic disorders in children is the disorder definition itself. The lack of adequate studies linking cardiovascular risk factors in childhood to disease in adulthood leaves pediatric definitions of metabolic disorders based on the distribution of cardiovascular measures in generally healthy children [12]. Therefore, thresholds are controversial. One approach is to model measures continuously, and rely on categorization only for clinical diagnosis [13, 14]. Tracking studies — defined as the maintenance over time of a relative position in the distribution of a variable [15] — have been analyzed mainly using thresholds to categorize the variables of interest, and stratify subjects into risk groups [16]. To avoid that, we propose to study the tracking of the rank values instead of the values themselves. That is, we aim to study whether the subjects with higher values at 4 years still have higher values at 8 years in terms of the variable distribution. With this goal, we consider data from the Infancia y Medio Ambiente (INMA)-Asturias cohort [17] and use quantile regression models [18]. This methodology allows to estimate the effect of an explanatory variable on any quantile of the outcome distribution, permitting the analysis of extreme values of the outcome without setting arbitrary thresholds [19].

For this reason, we aimed to apply this approach to assess whether having extreme values in the cardiovascular-related measures at 4 years is associated with having extreme values in the same cardiovascular-related measures at 8 years. We consider the following measures: triglycerides (TG), high-density cholesterol (HDL-c), atherogenic coefficient (AC), waist circumference to height ratio (WC/Height ratio), mean arterial pressure (MAP), and the homeostatic model assessment of insulin resistance (HOMA-IR).

## Materials and methods

### Study design

Study subjects were children participating in the INMA (Infancia y Medio Ambiente [Environment and Childhood]) Asturias cohort (north of Spain). Details can be found in previous studies [20, 21]. Briefly, between May 2004 and June 2007, pregnant women in their first trimester of pregnancy were recruited at the San Agustín University Hospital (Avilés) following a common protocol [17]. This hospital is a public health center with 436 beds which provides primary care and central, medical, and surgical services to a population of 144,875 inhabitants according to 2021 census [22]. The inclusion criteria were maternal age ≥ 16 years, singleton pregnancy, delivery scheduled at the referenced hospital, no assisted conception, and no communication handicap*.* Data were collected by trained professionals in several phases of follow-up: at first and third trimester of pregnancy, at birth, and at children’s ages 18 months, 4, and 8 years. Information was collected by medical registries, interview-based questionnaires with mothers, blood sample collection, and physical examinations of the children conducted by trained staff.

### Cardiovascular-related measurements

For this study we focused on cardiovascular-related measures that reflect well-established CVD risk factors in adulthood: central obesity, insulin resistance, dyslipidemia, and hypertension. These included WC/Height ratio for central obesity [23]; MAP for hypertension [24]; TG, HDL-c, and AC for dyslipidemia [25]; and HOMA-IR for insulin resistance [26].

#### Lipids

Lipids were measured at 4 and 8 years collecting non-fasting blood samples, obtained by antecubital venipuncture. Serum total cholesterol (T-c), TG, HDL-c, and low-density cholesterol (LDL-c) levels were determined using a Roche analyzer (Modular Analytics Serum Work Area, Mannheim, Germany). AC was calculated as the difference between T-c and HDL-c, divided by HDL-c. Lipids values are presented in milligrams per deciliter (mg/dL).

#### Anthropometry

At 4 and 8 years, trained staff measured children height and WC. Height was measured twice to the nearest 0.1 cm using a wall-mounted stadiometer after the participant removed their shoes. Waist circumference was measured to the nearest 0.1 cm at the children midpoint between the right lower rib and the iliac crest at the level of the umbilicus, using an inelastic nylon tape in a horizontal plane, and with children in a standing position. WC/Height ratio was calculated as waist circumference in cm divided by height in cm.

#### Blood pressure

Systolic blood pressure (SBP) and diastolic blood pressure (DBP) were measured using an automated oscillometric system (OMRON^®^) at children 4 and 8 years. After a 5-min rest period, between two and three consecutive measurements were taken, with children in a seated position and their right arm at rest at the heart level. The SBP and DBP averaged paired values were used and MAP was calculated as DBP + 1/3(SBP − DBP) [27]. Values are presented in millimeter of mercury (mmHg).

#### Blood glucose and insulin

Blood glucose and insulin levels were determined using the same Roche analyzer at children 4 and 8 years through collecting non-fasting blood samples, obtained by antecubital venipuncture. Glucose values are presented in milligrams per deciliter (mg/dL) and insulin values in microunits per milliliter (µU/mL). HOMA-IR was calculated as glucose multiplied by insulin and divided by 405.

### Potential confounding factors

The following parental characteristics were selected as potential confounders: maternal age at enrollment, maternal pre-pregnancy (BMI), paternal BMI, maternal educational level, maternal social class, maternal smoking during pregnancy, and parental CVD antecedents (neither parent has antecedents/one parent has at least one antecedent/both parents have at least one antecedent). Regarding pre-pregnancy BMI, the maternal height and pre-pregnancy weight were self-reported, both at the first-trimester visit. These values were used to calculate the pre-pregnancy BMI (in kg/m^2^). Paternal weight and height were reported by the mother at the first-trimester visit and were used to calculate paternal BMI. Questionnaires administered during the first and third trimester of pregnancy obtained information on maternal and paternal age and education, maternal country of birth, maternal and paternal occupation, and maternal smoking during pregnancy. Social class was defined according to the occupation during pregnancy of the mother or father, using a widely used Spanish adaptation of the International Standard Classification of Occupations coding system [28]. Parental CVD antecedent’s variable was reported by the mother in the first trimester of pregnancy. She was asked whether she or the father had been diagnosed with diabetes, heart disease, coagulation disorders, hypertension, or hypercholesterolemia and the responses were combined to create a categorical variable according to whether neither parent had any of them, whether one parent had at least one of them, or whether both parents had at least one of them. Children characteristics selected as potential confounders were age, height, weekly out-of-school physical activity time, and the mean of the daily energy intake. All of them were collected at the 4- and 8-year follow-ups. Week of gestation at delivery, birth weight, predominant breastfeeding duration, and sex were also considered. These information were collected from medical records, except for data on predominant breastfeeding duration, which were collected when the children were approximately 6 and 14 months old through questionnaires. Weekly out-of-school physical activity time was self-reported by mothers. The mean of the children daily energy intake was calculated based on validated food frequency questionnaires (FFQs) about children’s diet that were administered twice to the parents or care-givers of children over a 9-month period at 4 years, and over a 9–12-month period at 8 years. The FFQs were composed by 105 items at 4 years and by 46 items at 8 years. To explore the reproducibility of the FFQs, the nutrient and food group intake collected from the both FFQs at each age were compared, while validity was examined by contrasting the nutrient values from the FFQs and the average of three 24-h dietary recalls taken in this period, and also with the concentration of several vitamins in the blood (carotenoids, vitamin D, and α-tocopherol) [29, 30]. Nutrient values and total energy intake were calculated based on the US Department of Agriculture’s food composition tables and other published national sources. All questionnaires were conducted face-to-face by trained interviewers. The selection of these variables as potential confoundings was based on previous studies.

### Study population

**Fig. 1:**
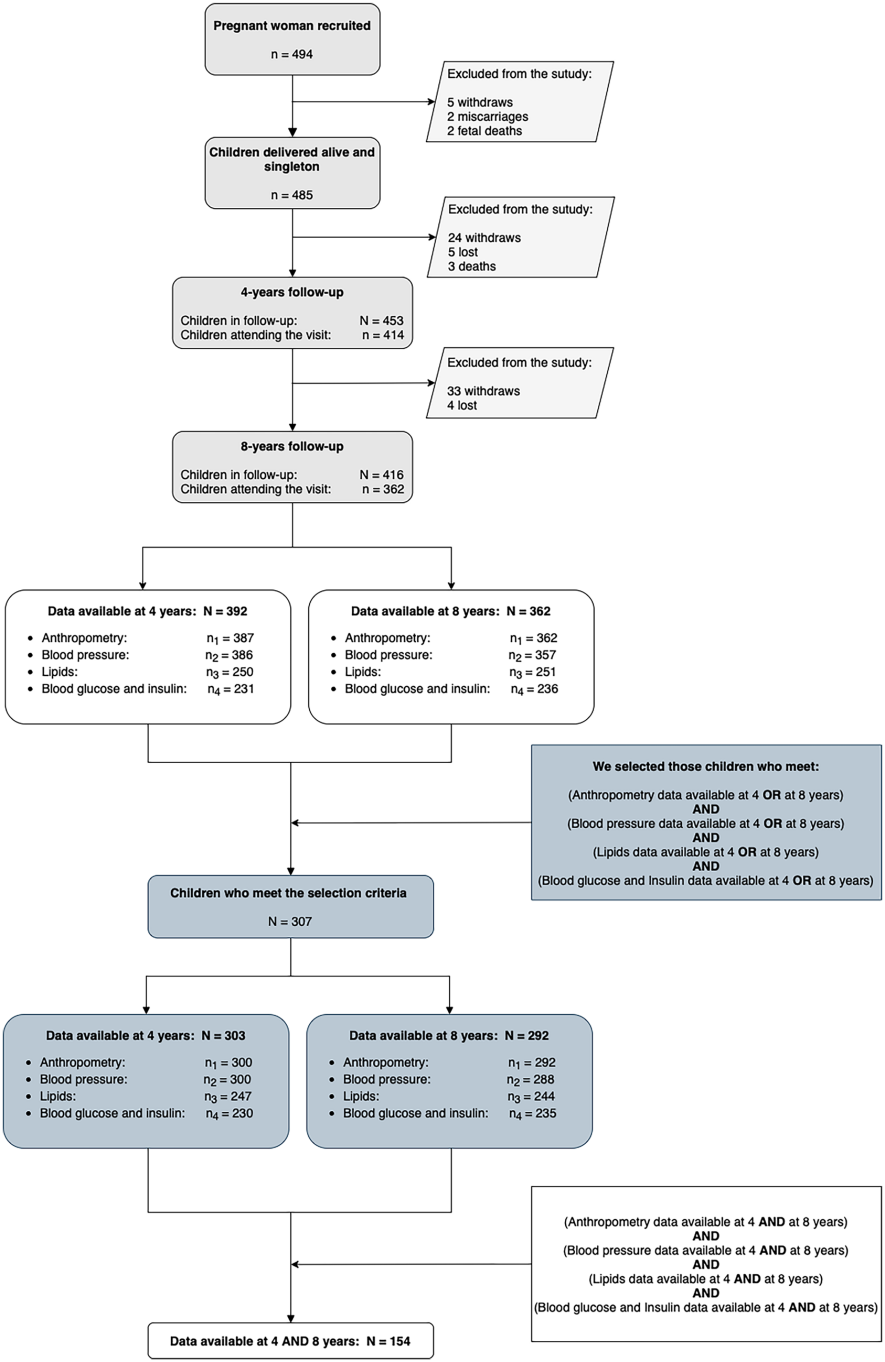
Flowchart of the study sample

Initially, 494 eligible women agreed to participate and, at birth time, 485 children were part of the study. At 4 years, 453 children continued in the follow-up and 91.4% of them attended to this follow-up visit. At 8 years, 416 children continued in the follow-up and 87.0% of them assisted to the follow-up visit. We limited the study to those 416 children who continued in the study at the 8-year follow-up. Of these 416 children, there were 392 with some measure of anthropometry, blood pressure, lipids, or glucose/insulin at 4 years and 362 at 8 years. Only 154 children had measurements of all variables involved in the study at 4 and 8 years. To optimize the use of the available information, we only excluded from the final sample those children who, for at least one of the cardiovascular-related measures involved in the study, had no data at neither 4 nor 8 years. The final sample was composed by 307 children. Figure 1 shows the flowchart of the study sample and the resulting sample size.

### Statistical analysis

Continuous variables were summarized by medians and interquartile ranges, and categorical variables by absolute and relative frequencies.

Crude and adjusted quantile regression models were performed to evaluate the association between the distribution of each cardiovascular-related measure at 8 years as the dependent variable, and the rank transformation — replacing the data by their corresponding ranks — of the same cardiovascular-related measure at 4 years as the independent variable. Quantile regression is a method used to evaluate the effects of exposures on the distribution of a continuous outcome [31]. It allows to assess whether the association between them differs for high-risk subjects (i.e., those at highest quantiles of the outcome) than for average subjects in the outcome distribution. To describe the effect of the independent variable on the cardiovascular-related measure distribution at 8 years, quantile sequence was estimated from 0.1 to 0.9. The models were fully adjusted with potentially confounding variables described in the “Potential confounding factors” section. Models were computed using the *quantreg* R package (version 5.94) [32] and standard errors were estimated using the “*xy*-pair” bootstrap method. To facilitate the interpretation of the regression parameters, the variables resulting from the rank transformation were expressed as percentiles. The reported parameters represent the effect on each quantile of the dependent variable of a 10-unit increase (1-decile) in the independent variable. The analysis was repeated with each cardiovascular-related measure at 8 years as the dependent variable, and the rank transformation of the same cardiovascular as the independent variable, but also including as independent variables the rank variables of other five remaining cardiovascular-related measures, adjusted by covariates (referred as the *complete model* in following sections).

All the analyses were performed after missing value multiple imputation in all the cardiovascular-related measures and the adjustment variables [33]. Under the missing at random (MAR) assumption, that our data suggest that this may be plausible, we applied multivariate imputation by chained equation (MICE) method with fully conditional specification using the mice R package (version 3.14.0) [34]. The results were pooled using Rubin’s combination rules [35].

The criteria used to select the final sample resulted in groups of children at 4 and 8 years with a complex structure between them, combining both independent and related measures. To test hypotheses about difference in means or proportions between these groups maintaining the original structure of the data and their relations, we use the general bootstrap algorithm (gBA) for hypothesis testing [36].

All the analyses were conducted using the R statistical software, version 4.2.1 (R Project for Statistical Computing). Statistical significance was considered at *p*-value < 0.05.

## Results

Following the inclusion criteria mentioned in the “Study population” section, a total of 303 children with data on at least one of the cardiovascular-related measures at 4 years were included in the analysis, and a total of 292 children who meet the same criterion were included at 8 years. The merging of these two subsamples results in total sample of 307 children (Table 1). There were 288 children present in both subsamples at the same time, 15 children had only in the 4-year but not in the 8-year subsample, and 4 children had only 8-year data but not 4 years. Due to the small difference between the subjects in each sample, there are no relevant differences in not age-related characteristics. Maternal median age at delivery was 32.9 and 33.1 years at each subsample, respectively, and more than 96% of the mothers were from Spain. Overall, 33.0% of mothers and 66.8% of fathers in the 4-year subsamples, and 32.9% of mothers and 66.2% of fathers in the 8-year subsamples, were overweight or obese (BMI ≥ 25 kg/m^2^). The average daily energy consumed increased from age 4 to age 8 (a median of 1618 and 1753 cal, respectively; *p*-value = 0.001), and the number of weekly hours of physical activity outside school was considerably reduced from 4 to 8 years (a median of 11.5 and 3.00 h, respectively; *p*-value < 0.001). This decrement is explained because at age 4, parents reported an average of 8.3 h per week of playing at home or in playground, and this activity is no longer reported at 8 years. Table 2 contains the summary of anthropometric, serum lipids, blood pressure, and glucose and insulin variables in the 4- and 8-year subsamples.

**Table 1:** Characteristics of the study sample, before multiple imputation

	**4 years (*n* = 303)**	**8 years (*n* = 292)**	***p*-Value**^a^
**Parental characteristics**			
Maternal age at delivery (years)	32.9 [30.4, 36.0]	33.1 [30.5, 36.0]	0.815
Maternal origin country			0.999
Spain	292 (96.4%)	282 (96.6%)	
Latin-American	6 (2.0%)	5 (1.7%)	
Europe	4 (1.3%)	4 (1.4%)	
Other	1 (0.3%)	1 (0.3%)	
Maternal level of education			0.996
Primary	47 (15.5%)	44 (15.1%)	
Secondary	140 (46.2%)	134 (45.9%)	
University	116 (38.3%)	114 (39.0%)	
Maternal social class			0.984
Upper I + II	72 (23.8%)	70 (24.0%)	
Middle III	63 (20.9%)	64 (22.0%)	
Low IV + V	167 (55.3%)	157 (54.0%)	
Maternal smoking during pregnancy			0.951
No	244 (84.4%)	233 (84.1%)	
Yes	45 (15.6%)	44 (15.9%)	
Maternal pre-pregnancy BMI (kg/m^2^)	23.9 [21.7, 28.1]	23.9 [21.6, 28.1]	0.803
Categorical maternal pre-pregnancy BMI^b^			0.946
Normal	203 (67.0%)	196 (67.1%)	
Overweight	72 (23.8%)	70 (24.0%)	
Obese	28 (9.2%)	26 (8.9%)	
Paternal BMI (kg/m^2^)	27.0 [24.6, 30.2]	27.0 [24.6, 30.2]	0.733
Categorical paternal BMI^b^			0.968
Normal	97 (33.2%)	95 (33.8%)	
Overweight	146 (50.0%)	142 (50.5%)	
Obese	46 (16.8%)	44 (15.7%)	
Parental cardiovascular antecedents			
Neither parent has antecedents	264 (87.1%)	253 (86.6%)	0.945
One parent has at least one antecedent	39 (12.9%)	39 (13.4%)	
Both parents have at least one antecedent	0 (0%)	0 (0%)	
**Child characteristics**			
Sex			0.979
Female	138 (45.5%)	134 (45.9%)	
Male	165 (54.5%)	158 (54.1%)	
Age (years)	4.40 [4.33, 4.53]	8.26 [8.08, 8.38]	< 0.001
Mean daily energy intake (calories)	1618 [1429, 1876]	1753 [1441, 2104]	0.001
Weekly out-of-school physical activity time (h)	11.5 [8.00, 16.0]	3.00 [2.00, 4.75]	< 0.001
Week of gestation at delivery	39.6 [38.6, 40.6]	39.6 [38.5, 40.6]	0.821
Predominant breastfeeding duration (weeks)	10.8 [0.00, 21.6]	10.7 [0.00, 21.6]	0.734
Birth weight (g)	3300 [3010, 3600]	3290 [3000, 3570]	0.879

Characteristics of the 303 children who have data on at least one of the cardiovascular measures involved in the analysis at 4 years of age and of the 292 children who meet the same criteria at 8 years of age. Continuous variables are summarized by medians and interquartile ranges, and categorical variables are summarized by absolute and relative frequencies

^a^The *p*-values were calculated using the general bootstrap algorithm for hypothesis testing (gBA) mentioned in the “Statistical analysis” section

^b^BMI was categorized according to WHO criteria: normal, BMI < 25 kg/m^2^; overweight, 25 kg/m^2^ ≤ BMI < 30 kg/m^2^; obese, BMI ≥ 30 kg/m^2^

**Table 2:** Cardiovascular-related measures of the study population, before multiple imputation

Measures	4 years (*n* = 303)	8 years (*n* = 292)
Weight (kg)	18.0 [16.7, 20.0]	29.6 [26.2, 34.3]
Height (cm)	106.0 [103.0, 109.0]	131.0 [127.0, 135.0]
BMI (kg/m^2^)	16.0 [15.3, 17.2]	17.3 [15.8, 19.4]
Categorical BMI^a^		
Normal	236 (77.9%)	196 (67.1%)
Overweight	41 (13.5%)	68 (23.3%)
Obese	26 (8.58%)	28 (9.59%)
Waist circumference (cm)	53.5 [50.5, 56.0]	63.5 [58.9, 69.0]
Waist circumference/Height ratio	0.50 [0.48, 0.53]	0.48 [0.46, 0.52]
Triponderal index (kg/m^3^)	15.2 [14.4, 16.3]	13.3 [12.1, 14.5]
Systolic blood pressure (mmHg)	99.0 [90.0, 105.0]	107.0 [100.0, 114.0]
Diastolic blood pressure (mmHg)	60.0 [54.0, 66.0]	67.0 [60.8, 72.0]
Categorical blood pressure^b^		
Normal level	218 (72.7%)	104 (63.9%)
Monitoring level	35 (11.7%)	64 (22.2%)
Intervention level	47 (15.7%)	40 (13.9%)
Mean arterial pressure (mmHg)	73.0 [66.7, 77.7]	80.0 [74.2, 84.7]
Total cholesterol (mg/dL)	164.0 [147.0, 178.0]	165.0 [150.0, 183.0]
High-density lipoprotein cholesterol (mg/dL)	57.0 [47.5, 63.0]	68.0 [59.8, 80.0]
Low-density lipoprotein cholesterol (mg/dL)	92.0 [74.0, 106.0]	81.5 [65.0, 98.0]
Triglycerides (mg/dL)	71.0 [55.0, 94.0]	64.0 [47.2, 87.8]
Atherogenic coefficient	1.92 [1.50, 2.46]	1.39 [1.02, 1.78]
Glucose (mg/dL)	86.0 [81.8, 91.0]	86.0 [82.0, 90.0]
Insulin (µU/mL)	5.80 [3.10, 10.60]	7.75 [5.10, 13.6]
HOMA-IR	1.17 [0.64, 2.37]	1.69 [1.05, 2.88]

^a^BMI was categorized according to IOTF criteria

^b^Categorical blood pressure was categorized using age-, sex-, and height-specific thresholds provided by the IDEFICS study. Children were classified in the monitoring or the intervention level if they had SBP or DBP above the age-, sex-, and height-specific corresponding threshold

Additional file 1 shows the percentage of imputed data for each variable over the overall sample. Lipids, glucose, and insulin measurements had the highest percentage of missing data (ranging from 19.2 to 25.1% at 4 years and from 19.9 to 23.1% at 8 years). Missing data in anthropometric and blood pressure measurements ranged from 2.0 to 6.2%.

### Triglycerides

**Fig. 2:**
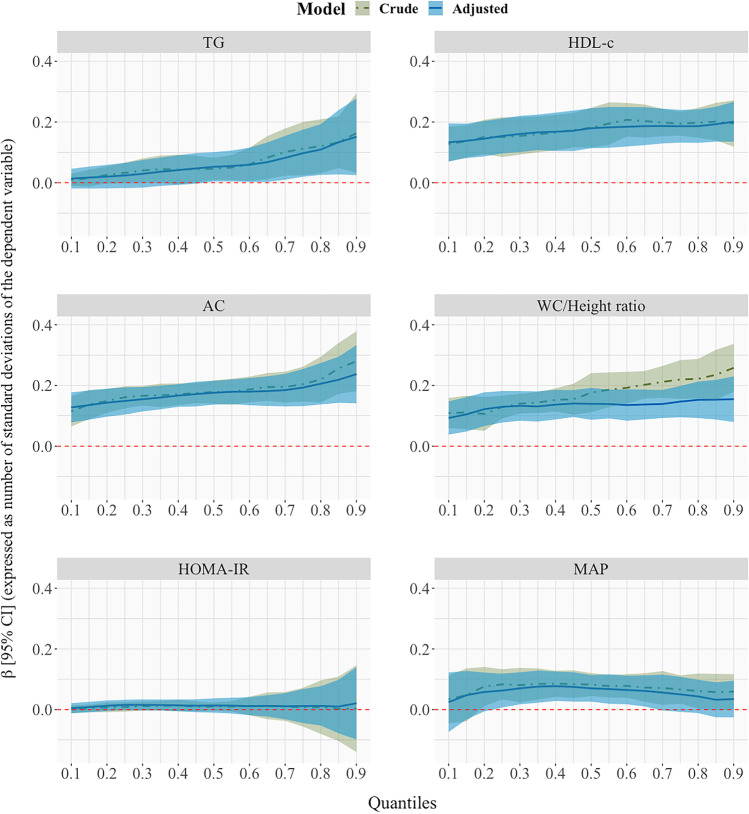
Quantile regression models with cardiovascular-related measure at 8 years as dependent variable and the rank variable of the corresponding cardiovascular-related measure at 4 years as the independent variable, for the quantiles between 0.1 to 0.9, with increments of 0.05, adjusted for maternal age at delivery, maternal level of education, maternal social class, maternal smoking during pregnancy, maternal pre-pregnancy body mass index, paternal body mass index, parental cardiovascular antecedents, child sex, child mean daily energy intake at 4 and 8 years, child weekly out-of-school physical activity time at 4 and 8 years, week of gestation at delivery, weeks of predominant breastfeeding, and child height at 4 and 8 years. Coefficient estimated are calculated with the independent variables in terms of percentiles and they represent the effect on the dependent variable for each 1-decile increase in the independent variable. They are expressed in terms of number of standard deviations of the dependent variable to homogenize the Y-axis scales

**Fig. 3:**
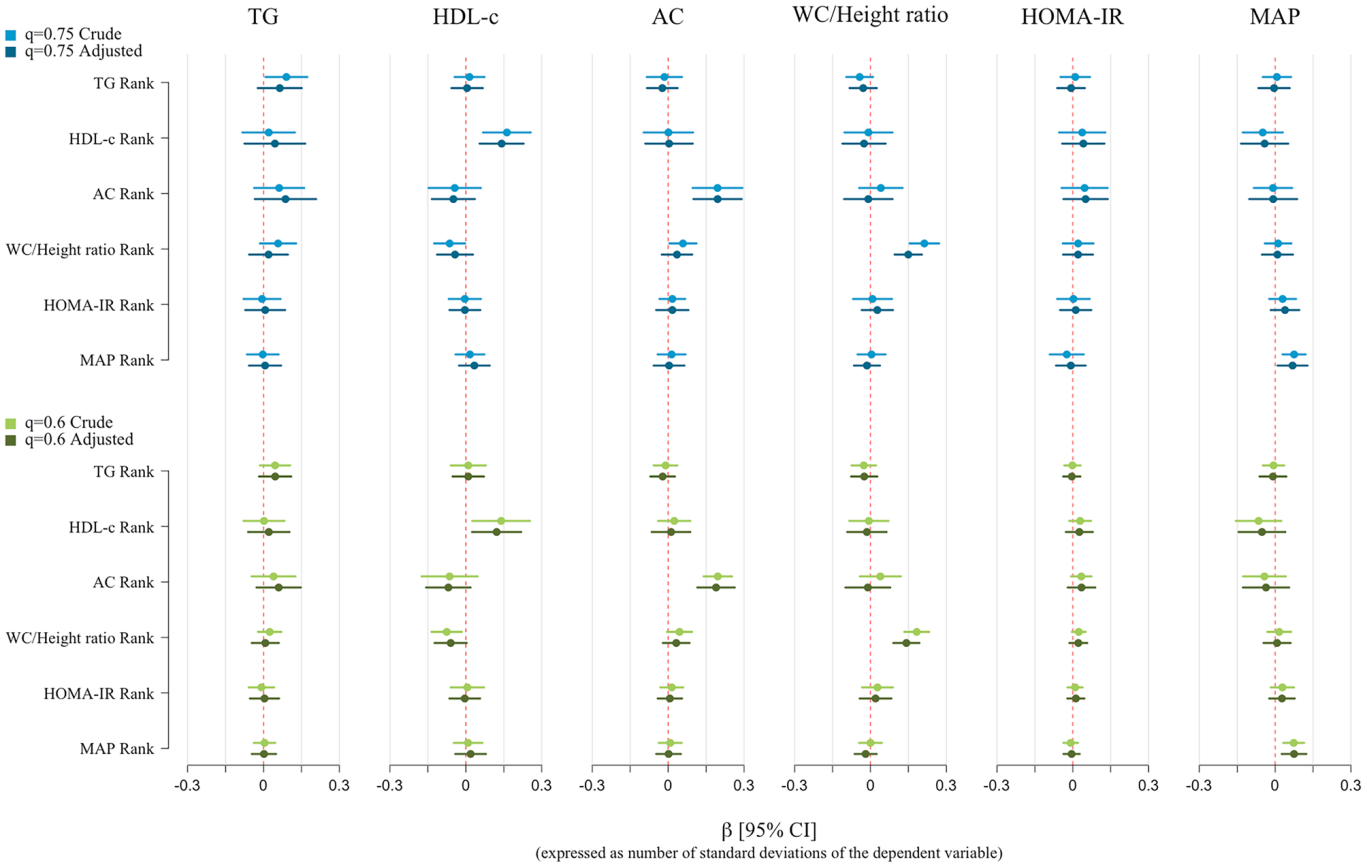
Quantile regression models with each cardiovascular-related measure at 8 years as dependent variable and the rank of all the cardiovascular-related measures at 4 years as the independent variables, for the quantiles 0.60 and 0.75, adjusted for maternal age at delivery, maternal level of education, maternal social class, maternal smoking during pregnancy, maternal prepregnancy body mass index, paternal body mass index, parental cardiovascular antecedents, child sex, child mean daily energy intake at 4 and 8 years, child weekly out-of-school physical activity time at 4 and 8 years, week of gestation at delivery, weeks of predominant breastfeeding, and child height at 4 and 8 years. Coefficient estimated are calculated with the independent variables in terms of percentiles and they represent the effect on the dependent variable for each 1-decile increase in the independent variable. They are expressed in terms of number of standard deviations of the dependent variable to homogenize the X-axis scales

Figure 2 shows the estimated quantile regression parameters for each rank cardiovascular-related measure at 4 years, on the distribution of the same cardiovascular-related measure at 8 years, for all quantiles. We observe a positive association between TG rank at 4 years and TG distribution at 8 years above 0.5 quantile. The magnitude of the association was stronger in the upper part of the distribution: 1-decile increase in child’s rank at 4 years related to an increase of 2.28 mg/dL (95% CI: 0.13, 4.43) in the 0.6-TG quantile at 8 years compared to an increase of 5.82 mg/dL (95% CI: 1.00, 10.65) in the 0.9-TG quantile at 8 years (Additional file 2). In the complete model (Fig. 3), 1-decile increase at 4 years has an increase effect of 1.77 mg/dL (95% CI: − 0.68, 4.23) in the 0.6 quantile compared to a 2.47-mg/dL (95% CI: − 0.88, 5.83) increase effect in the 0.75 quantile (Additional file 3).

### High-density lipoprotein cholesterol

The association between HDL-c rank at 4 years and HDL-c distribution at 8 years was positive across all quantiles (Fig. 2). A gradual increase was observed in the upper part of the distribution with an increase of 2.68 mg/dL (95% CI: 1.75, 3.62) for the 0.6 HDL-c quantile at 8 years and of 2.93 mg/dL (95% CI: 1.98, 3.87) for the 0.9 HDL-c quantile at 8 years (Additional file 2). Associations in the complete model (Fig. 3) were lower than in the individual model but the overall trends were similar.

### Atherogenic coefficient

The association between the AC rank at 4 years and AC distribution at 8 years also was positive (Fig. 2). The size of increase was greater at the highest part of the AC distribution (an increase of 0.11; 95% CI: 0.09, 0.14) in the 0.6 quantile vs. an effect of 0.15 (95% CI: 0.09, 0.21) in the 0.9 quantile (Additional file 2). Results were similar in the complete model (Fig. 3; Additional file 3).

### Waist circumference to height ratio

A positive association was observed between WC/Height rank at 4 years and WC/Height distribution at 8 years (Fig. 2). The crude model shows a positive trend in the effect size as the quantile increases, evidenced by the positive slope of the plot in Fig. 2 (effect of 0.010 (95% CI: 0.006, 0.013) on the 0.6 WC/Height quantile at 8 years vs. an effect of 0.014 (95% CI: 0.010, 0.018) on the 0.9 WC/Height quantile at 8 years; Additional file 2). In the adjusted model, the effect sizes were smaller and generally constant in all quantiles (increase of 0.007 (95% CI: 0.005, 0.010) in the 0.6 WC/Height quantile vs. an increase of 0.008 (95% CI: 0.004, 0.012) in the 0.9 WC/Height quantile at 8 years; Additional file 2). This difference in the trend of the crude and adjusted model is mainly due to the adjustment for maternal BMI and educational level. The complete model (Fig. 3) produced similar results.

### HOMA-IR

No clear association was found between HOMA-IR rank at 4 years and HOMA-IR distribution at 8 years (Fig. 2) (effect size of 0.037 (95% CI: − 0.058, 0.131) in the 0.6 HOMA-IR quantile vs. 0.067 (95% CI: − 0.312, 0.445) in the 0.9 HOMA-IR quantile at 8 years; Additional file 2). This also was seen in the complete model (Fig. 3).

### Mean arterial pressure

The association of MAP rank at 4 years on MAP distribution at 8 years was positive, but only statistically significant between 0.3 and 0.6 quantiles (Fig. 2). Similar results were observed in the complete model (Fig. 3).

## Discussion

This study found a positive association between the relative position of children at 4 years in the HDL-c, AC, and WC/Height distributions and all the quantiles of the same variable at 8 years. For TG distribution, it was found a positive association between the relative position at 4 years and the quantiles above 0.5 at 8 years, but which is not observed in the model adjusted for the rest of the cardiovascular-related measures. The stronger associations in the upper parts of the distribution in terms of standard deviations of each variable were found for HDL-c and AC outcomes. For AC, the more extreme the children’s values at 8 years, the greater the effect of the association. This trend is also observed for TG, although the effect is not statistically significant. No conclusive association was found for either HOMA-IR or MAP outcomes.

Our findings for HDL-c and AC suggest serum lipid track among children between the ages of 4 and 8 years. These results are in line with those found in children from two different Japan rural areas: one area reported tracking of serum lipids (specifically in T-c, HDL-c, and AC) between 8 and 12 years [37] and the other reported strong T-c tracking in children aged 6–7 after 9 years of follow-up [38]. Previously, The Muscatine Study observed T-c and TG tracking in children between 5 and 12 years after follow-ups of 2, 4, and 6 years [39]. The Bogalusa Heart Study showed tracking of serum lipids in 5-year-old children after a follow-up of 9 years [40]. These studies categorized serum lipids and evaluated which percentage of children remained in the highest category after the follow-up, which does not allow to observe differences in tracking within the highest-risk category itself. Using our quantile regression approach, we were able to observe that for AC the magnitude of tracking was stronger as the relative position at 8 years of age increases, showing the possible difficulty that children with abnormal values at 4 might have in normalizing them in future years. This is remarkable because AC (the ratio of non-HDL-c to HDL [25]) is clearly related to higher risk of CVDs in adulthood [41, 42]. Whereas higher HDL-c had similar tracking levels in all quantiles and higher HDL-c has unclear association with CVD risk [43].

Tracking of measures related to obesity have been widely studied in childhood and adolescence. The majority of studies use BMI as a marker of obesity [37, 44–48]. Increasingly studies are focusing on other anthropometric measures such as WC, WC/Height ratio, or skinfolds [49–51] and report the presence of tracking, consistent with our findings, in a variety range of ages but mostly between childhood and adolescence. Some of these studies find tracking among the same age ranges that were considered in this study [46, 48, 51], although the evaluation approach makes difficult to compare effect magnitudes. Hayes et al. [46] evaluated tracking of BMI in seven follow-up visits between 2–3 and 16–17 years and reported that the tracking magnitude was lower between 2 and 7 years than at later ages. A meta-regression analysis reported stronger BMI tracking after the age of 7 [52]. This suggests steeper tracking of central obesity than observed in our study for children of older ages. In the crude model, we observed higher tracking in children with a high-risk position in the distribution at 8 years. But when we adjusted for maternal pre-pregnancy BMI, and maternal educational level, the association became very similar across all the quantiles of the distribution: higher maternal BMI is associated with a tracking increasing effect in the highest quantiles, and higher maternal educational level is associated with a decreasing effect in the highest quantiles. Other studies reported similar findings with maternal pre-pregnancy BMI and measures related with obesity, and with blood pressure as well [53]. We only have observed this effect in measures related with obesity. Several studies have reported the influence of socioeconomical inequalities in BMI tracking, using parental educational level [54], parental socioeconomic position [46], or a combination of parental educational level, household income, and occupation [48]. We have observed this effect with maternal educational level but not with socioeconomic status.

Neither the MAP nor HOMA-IR showed a relevant association between the relative position of children at age 4 years and the relative position at age 8 years in the upper part of the distribution. As with serum lipids, there are few studies that analyzed the tracking of these measures in childhood and adolescence, rather than in adulthood. In our study, MAP is used as a blood pressure index to try to capture the effect of systolic and diastolic pressure using a single measurement. However, we also examined SBP and DBP separately (data not shown), yielding identical findings and conclusions as those obtained using MAP. Existing studies predominantly employ SBP and DBP and most of them report weak or poor blood pressure tracking [37, 39, 55], in line with the results we obtained here. One exception is the study by Sánchez-Bayle et al. [56] that reported considerably higher level of blood pressure tracking in a school-aged population. For measures related to insulin resistance, two studies examined tracking between 8 and 21 years [57], and between 10 and 17 years [58], with disparate results. Joshi et al. [57] reported moderate tracking of the HOMA-IR but no tracking of fasting insulin or glucose measures, while Wang et al. [58] reported no tracking in the HOMA-IR but tracking of fasting glucose. It should be pointed that in longitudinal studies a change in the behavior of the subjects can arise based on the knowledge of the results and the recommendations given in this regard, influenciating the tracking effect. Despite the limitation this may imply, these recommendations make it possible to reverse trends that would be more difficult in adulthood.

Numerous studies have reported interrelations between cardiovascular risk measures including markers of obesity, blood pressure, insulin resistance, and lipids, although these relations and the pathways explaining them are not clear yet [59, 60]. Therefore, in our study, the analyses were repeated including the cardiovascular-related measures as independent variables altogether in our models to observe whether any were acting as confounding factors for each other. The results of the individual analysis were remained, although with a general attenuation of the magnitude of the effects.

To our knowledge, this is among the first studies to address the analysis of cardiovascular measure tracking in children using quantile regression. Only one prior study conducted the analysis of BMI tracking between childhood and adulthood using this statistical technique [54]. This approach allows the introduction of several adjustment variables and exploration of the effects of different variables at the same time, as well as avoids using thresholds, always controversial in pediatric ages. Among other strengths of this study is that by using quantile regression and the independent variables in terms of their ranges allows to control for age-dependent variation in the effects observed for measures as HDL-c or TG.

This study has also some limitations. The sample size is moderate/small, with the consequent loss of power in the analysis. This is an exploratory study, in which numerous hypotheses are tested (different quantiles and different results), so that multi-testing problems arise, making problematic to calculate the statistical power of the study. Some of the variables were self-reported by the children’s parents. Other variables that have shown to influence the associations evaluated here have not been included such as children diet quality [61], maternal diet quality during pregnancy and breastfeeding [62], or pre-eclampsia [63]. It should also be noted that blood samples were collected under non-fasting conditions. It is unclear to what extent glucose and insulin levels might be biased due to prior caloric intake [64]. However, in non-diabetic subjects, the HOMA-IR would not be expected to show large variations. If blood glucose is higher due to previous intake, insulin also raises its levels, and therefore, the ratio between them will be similar. No blood glucose levels suggestive of diabetes have been detected in our sample, so we expect the HOMA-IR to be similar to that under fasting conditions. Regarding to lipids, the use of non-fasting samples is already recommended, except in unusual cases that do not apply to our sample [65–67]. On the other hand, most studies evaluate tracking between longer periods, and extreme values of cardiovascular-related measurements in childhood have shown to have an age-dependent impact on adult cardiovascular health, being predictive of subclinical atherosclerosis from the age of 9 [68]. Yet, it is still relevant to know the tracking of these measures throughout early childhood and adolescence more as a continuum, as long-term effect of childhood exposures on adult health is likely cumulative [11].

## Conclusions

Our study found tracking between 4 and 8 years of age at the highest quantiles of the distribution of cardiovascular-related measures established as adult markers of dyslipidemia and central obesity (specifically for HDL-c and AC for dyslipidemia and WC/Height ratio for central obesity). The results indicated that for AC distribution tracking appears to be stronger at higher quantiles, suggesting the difficulty of normalizing their extreme values. These findings can help determine what cardiovascular-related measures could be the targets of screening and monitoring in children.

### Supplementary Information

Below is the link to the electronic supplementary material.

Supplementary file1 (XLSX 11 KB)

Supplementary file2 (XLSX 12 KB)

Supplementary file3 (XLSX 13 KB)

## Data Availability

The data and computing code are available for replication from the corresponding author on reasonable request.

## Additional information

The CHAMP statement and The STROBE guideline were used to ensure the reporting of this observational study [69, 70].

## Abbreviations

AC: Atherogenic coefficient
BMI: Body mass index
CVDs: Cardiovascular diseases
DBP: Diastolic blood pressure
gBA: General bootstrap algorithm
HDL-c: High-density cholesterol
HOMA-IR: Homeostatic model assessment of insulin resistance
LDL-c: Low-density cholesterol
MAP: Mean arterial pressure
MAR: Missing at random
MICE: Multivariate imputation by chained equations
SBP: Systolic blood pressure
T-c: Total cholesterol
TG: Triglycerides
WC: Waist circumference
WC/Height: Waist circumference to height ratio
